# A Case Series of Life-Threatening Hemorrhagic Events in Patients with COVID-19

**DOI:** 10.1007/s12262-021-02879-y

**Published:** 2021-05-08

**Authors:** Abbas Hajian

**Affiliations:** Department of General Surgery, Medical College, Kashan, Iran

**Keywords:** Antithromobotic, Anticoagulation, Bleeding, COVID-19, Hematoma

## Abstract

Since venous microthrombotic and thromboembolic events in end organs have been pathophysiologically confirmed as a component of thrombo-inflammatory cascade in COVID-19 syndrome, anticoagulant prescription with prophylactic or therapeutic goal is recommended. Different guidelines for the above are introduced; however, there is no general consensus on any neither the type of anticoagulant nor for the dosage and duration of prescription. In our medical center, adopted internal guideline was considered for patients COVID-19. We consulted patients with COVID-19 who suffered from concurrent hematoma. Appropriate surgical approach was considered. Finally autopsy study was performed for patients. In this article, we presented a series of seven SARS-CoV-2 confirmed cases faced with bleeding complication following initiation of anticoagulation protocol. The rectus sheath hematoma with extension to pelvic and/or retroperitoneal space, even involving bowel mesentery was seen most commonly. Despite receiving appropriate surgical care, all seven cases died. Finally, in all cases, autopsy studies revealed no evidence for confirmation of DIC/SIC or organ failure as the reason of death although pulmonary involvement with SARS-CoV-2 and bleeding phenomena were approved. The nature of the COVID-19 syndrome makes patients vulnerable to hemorrhagic events following anticoagulant administration which relatively causes or accelerates patient’s expiration.

## Introduction

From last December 2019, the novel beta corona virus [COVID-19] was introduced with respiratory symptoms until the March 2020 that the World Health Organization [WHO] announced a global viral pandemic [1, 2]. The virus has diverse invasive behaviors and poor response to human immune system which leads to aggressive colonization, different organ involvement, recurrence, and death. Recent knowledge about pathophysiology of the disease revealed thromboinflammatory accidents at microvascular level of body systems including the lungs, central nervous system, heart, kidneys, liver, stomach, bowels, and etc. [3]. The virus binds to ACE-2 receptor at endothelial surface and activates infectious-related coagulopathy cascade [3]. Injured endothelium is then affected by blood flow stasis and thrombotic events. The primary result is microthrombosis formation that if it does’t restrained would end to target organ failure. Systemic response to this thromboinflammatory reaction encounters the victim to venous thromboemboli [VTE], deep vein thrombosis [DVT], pulmonary thromboemboli [PTE], acute coronary syndrome [ACS], brain stroke, sepsis-induced coagulopathy [SIC], and disseminated intravascular coagulation [DIC] with increased fibrinogen, d-Dimer, IL-6, von-Willebrand factor, and decrease in antithrombin and lymphocyte count [1–6]. Regarding these data, anticoagulation therapies have became prominent among inpatient and particularly ICU admitted subjects with COVID-19 disease [1, 3, 5, 6]. However, there is no general consensus yet about details of anticoagulation prescription. Unfractionated heparin [UFH], LMWH [enoxaparin], factor X inhibitor [fondaparinux, apixaban, rivaroxaban], direct thrombin inhibitor [dabigatran], antiplatelet (aspirin), and warfarin were utilized [1, 3, 5]. Although physicians insist on prescribing anticoagulants in medical centers based on presented guides, it is not clear that how these drugs would affect the patient to survive from thrombotic events. Hence, it seems important to not order anticoagulants for every SARS-CoV-2 positive while risk of hazardous complication including mortal hemorrhage could be relatively significant. Following toactivation of anticoagulation guideline for inpatient COVID-19 sufferer in our hospital we were called as general surgeon to consult some patients developed hematoma. Considering the latter we presented the cases with unpredictable course and outcome of the disease. Current article was written in lined with the SCARE 2020 guideline [7].

## Patients and Methods

### Diagnostic and Therapy Approach

Every patient who was suspicious for the SARS-CoV-2 initially admitted to particular ward equipped specifically for the clinical statusaccordingly. After receiving primary care, computed tomography [CT] scanning study of lungs and also polymerase chain reaction [PCR] test for SARS-CoV-2 from oropharyngeal secretion was performed individually. If the evaluations confirmed the COVID-19 pneumonia, patient then was transferred to COVID-19 ward/ICU and received proper therapy based on clinicaljudge for severity of the disease.

### Anticoagulant Guideline

According to our center instruction for VTE prophylaxis/treatment in patients with COVID-19, following guideline was ordered:

As Table 1 shows, every patient who admitted in hospital because of involvement with SARS-CoV-2 underwent anticoagulant prophylaxis or therapy. In case of UFH prescription, dosage titration was accordingly conducted based on activated partial thrombin time [aPTT] measurement. For all categories of patient condition daily, Acetylsalicylic Acid [ASA] 80 mg was prescribed if no contraindication was identified including liver and/or renal failure (Crcl < 15 mL/min) and/or ongoing GI bleeding.

**Table 1:** Guideline for VTE prophylaxis among COVID-19 patients

	Patient condition	Prescribed drug dose		Choice	Alternative
Inpatient	Severe^1^ ***to*** Critical^2^	Intermediate ***to*** therapeutic		Enoxaparin(SC): 40 mg (Crcl ≥ 30 mL/min) and 30 mg (Crcl: 15–30) daily ***to*** 1 mg/kg BID (Crcl ≥ 30) and 1 mg/kg daily (Crcl: 15–30)	UFH: 7500 IU TID(SC) ***to*** 1000 IU(IV) per hour
	Mild^3^ to Moderate^4^	Low		Enoxaparin(SC): 40 mg (Crcl ≥ 30) and 30 mg daily (Crcl: 15–30)	UFH(SC): 5000 IU BID to TID
Outpatient	Discharged or Not admitted	Low	If high risk^5^ for VTE and low risk for bleeding	Enoxaparin(SC): 40 mg (Crcl ≥ 30) and 30 mg daily (Crcl: 15–30)	Rivaroxaban(PO): 10 mg daily or Apixaban(PO): 2.5 mg BID
		None	If low risk for VTE	–	–

^1^Pneumonia + one of following: severe respiratory distress, RR > 30/min, SO2 < 90% in room air

^2^ICU admitted

^3^Respiratory symptoms except for radiologic signs for viral pneumonia or hypoxia

^4^Pneumonia (fever, cough, dyspnea, tachypnea) with SO2 ≥ 90% in room air

^5^History of VTE, active cancer, COPD, bed-ridden, valvular disease, atrial fibrillation, and d-dimer > 2000 ng/mL before discharge

## Case Presentation

### Case 1

A 26-year-old female with no past medical or surgical history, complained of a 3 days body pain, fever and chill, dyspnea of exertion, dry cough, and fever. In physical examination, blood pressure [BP] 135/75 mmHg, heart rate [HR] 110/min, respiratory rate [RR] 24/min, oral temperature [OT] 38.7 °C, and BMI 24 kg m^−2^ were registered. She was agitated and tachypneic with bilateral decreased respiratory sound [RS] in both lung bases with SO_2_:88% in room air. The CT study revealed viral pneumonia. She was admitted in ICU to receive therapy but not intubated. Subcutaneous enoxaparin was started 40 mg BID in combination with daily oral ASA 80 mg for her critical condition. On the day 5th of admission surgical consult was requested because of rectus sheath hematoma. We visited the patient. She was hemodynamically stable. Physical examination revealed a dense tender mass in right lower abdomen with no sign of peritonitis. Hemoglobin [Hb] level diminished by 1.5 g dL^−1^ during 24 h of hematoma diagnosis and INR was in normal range. Focused abdominal wall ultrasonography [US] manifested a heterogeneous hypoechoic 74 × 29 × 62 mm [about 70 cc] region in right rectus muscle sheath confirmed hematoma diagnosis. Enoxaparin and ASA were held and without packed blood cell [PBC] infusion patient underwent serial examination. She died 10 days after hematoma formation. Following US a day prior to expiration showed that the size of hematoma had been increased to 90 cc. Autopsy study showed no evidence of DIC, SIC, or organ failure. It confirmed presence of COVID-19 pneumonia and presented hematoma.

### Case 2

A 70-year-old woman with past medical history of ischemic heart disease, type II diabetes mellitus [DM], and BMI 32.3 kg m^−2^ with respiratory distress was transferred to the COVID-19 bay by the EMS. At first visit BP 110/60 mmHg, HR: 94/min, RR: 28/min, OT 39 °C, and SO_2_ %90 in room air were determined. Bilateral fine crackle was heard. The lung CT study was compatible with COVID-19 pneumonia and PCR test later confirmed the diagnosis too. She was admitted to ICU with severe disease status and underwent subcutaneous UFH 5000 IU three times a day in combination with daily oral ASA 80 mg for VTE prophylaxis. On the 6th day of admission, surgical consult was requested for abdominal pain. We visited the patient in ICU. She was hemodynamically stable. Abdominal examination revealed a tender mass unilaterally in lower abdomen sounded for the sheath of right rectus muscle hematoma. In abdominal US mild to moderate interloop fluid was seen in combination with a heterogeneous hypoechoic 73 × 70 × 22 mm [about 60 cc] lesion in favored with rectus sheath hematoma [RSH] diagnosis. Therefore, the UFH was held. The Hb level fell down 3.1 g dL^−1^ and was measured 9.8 g dL^−1^. PTT and INR were85 and 1.5 respectively on the day of hematoma diagnosis and did not rise in following evaluation. Patient was closely observed without PBC infusion. She died after 18 days of admission when examination showed near normal abdominal exam but about 110 cc of remained hematoma in controlled US. Autopsy study was otherwise normal except for presence of pulmonary involvement and stated hemorrhage.

### Case 3

A 58-year-old woman whose CT and PCR test for SARS-CoV-2 were positive underwent hospitalization in a non-ICU ward. She claimed that she had fever, cough, dyspnea of exertion, fatigue, and orthopnea from 2 days prior to admission. Registered data at her first visit was included of BP 150/90 mmHg, HR 83/min, RR 22/min, OT 38.2 °C, SO_2_ 91%, and BMI 38.6 kg m^−2^ with no specific change for RS in lungs. Past medical and surgical history was unremarkable except for appendectomy. Her disease severity was considered moderate and underwent subcutaneous UFH 5000 IU TID in addition to daily oral ASA 80 mg tablet. On the 2nd day of admission, we were called for surgical consult for expanding hematoma and painful movement of right upper limb and axilla. We identified total right upper limb hematoma and ecchymosis from shoulder joint superiorly to the wrist and dorsum of the hand inferiorly. Ipsilateral axillary region ecchymosis with extension to lateral breast border anteriorly, ribs 7–8 interspace inferiorly, and medial border of the scapula posteriorly was observed. History of recent trauma was negative. The right arm was tender and the skin was in tension. Distal arterial pulses at the wrist were palpated and no evidence of compartment syndrome was signed. Active limb movement was painful. Focused regional US showed a heterogeneous hypoechoic 33 × 20 × 18 mm (about 40 cc) hematoma formation in biceps brachii muscle. Anticoagulants were held. Conservative management with elevation of the extremity and physical therapy was regarded. Normal range for either Hb or for PTT/INR level maintained. After 9 days of admission patient was expired with extension of both right sided hematoma to 130 cc in controlled US with the regional ecchymosis. Autopsy study was revealed pulmonary involvement with COVID-19 and aforementioned bleeding with no extra sign of any other organ failure.

### Case 4

A 51-year-old woman suffered from generalized body pain, at rest dyspnea, productive cough, and fever from 2 days ago was admitted to the ICU. She had past history of hypertension and was under losartan-hydrochlorothiazide 50/12.5 mg BID. Prior trans-abdominal hysterectomy was mentioned. Presence ofSARS-CoV-2 was positive based on CT and PCR studies. Clinical findings were included BP 140/85 mmHg, HR 96/min, RR 25/min, OT 39.5 °C, SO_2_ 87%, BMI 34.7 kg m^−2^, and decreased RS in both of the lungs. The subject was considered with critical condition and given subcutaneous enoxaparin 60 mg BID in combination with oral ASA 80 mg per day. We were summoned for patient’s abdominal distension on the 7th day of admission to rule out surgical diagnoses. She lied on bed and tended to be immobile. Hemodynamic indexes were stable. In physical examination a distended abdomen with diminished bowel sounds, generalized mild tenderness, and evidence for peritonitis due to periumbilical rebound tenderness were remarkable. Abdominal CT study revealed a heterogeneous 121 × 83 × 70 mm (about 382 cc) hematoma of left rectus sheath that extended to ipsilateral pelvic fossa in addition to increased right rectus sheath thickness that was compatible forpresence of bilateral RSH. According to reverse Hb levels from 11.4 to 6.6 mg dL^−1^, PTT>120, and INR 1.4, three units of the crossed match PBC and 8 units of the fresh frozen plasma [FFP] were infused. Enoxaparin and ASA were also held. Patient was expired in the course of the primary disease after 13 days. Controlled US exam manifested about 430 cc of collected hematoma. Autopsy study declared no DIC, SIC, or any organ failure except for bilateral pulmonary involvement and above bleeding.

### Case 5

A 44-year-old woman with prior chemoradiation therapy following left breast mastectomy about 8 years before her recent SARS-CoV-2 confirmation was admitted to hospital. She complained of fever, dyspnea, cough, dizziness, and illness from a day before admission. Initial physical exam revealed BP 100/60 mmHg, HR 101/min, RR 33/min, OT 39.8 °C, SO_2_ 91% in room air, BMI 28.5 kg m^−2^, and fine crackles especially in the right lung. She underwent anticoagulant for severe disease. Subcutaneous enoxaparin 60 mg in addition to daily oral ASA 80 mg was prescribed. On the 6th day of admission, surgical consult was requested for abdominal pain and tenderness. We visited the patient while she was lied on bed with stable hemodynamic values who tried to prevent cough because of following abdominal pain. Abdominal examination manifested a tender mass-like lesion in the left side which was extended symmetrical to rectus muscle burden and was accompanied with diffused rebound tenderness. Abdominal US described a heterogeneous hypoechoic 82 × 66 × 23 mm (about 64 cc) hematoma in left rectus sheath with concurrent 200 × 78 × 41 mm (about 347 cc) hematoma in preperitoneal space. CT confirmed the diagnosis with no synchronous intraperitoneal lesion. The Hb decreased 2.2 g dL^−1^ and the INR level had no change. Both the enoxaparin and ASA was held. Patient did not receive PBC or FFP. Serial examination and controlled US showed neither progression nor meaningful reabsorption of the hematoma in next 5 days. Patient died after 6 days of hematoma diagnosis while prepared for right upper extremity fasciotomy due to initiation of signs of compartment syndrome. Autopsy study was confirmed lung involvement and aforementioned bleeding. No sign of DIC or SIC was seen.

### Case 6

A 72-year-old man was diagnosed as a SARS-CoV-2-positive subject. He was admitted to the hospital with moderate disease severity. Patient’s characteristics were initially included BP 130/90 mmHg, HR 109/min, RR 30/min, OT 37 °C, SO_2_ 90% in room air, BMI 27.4 kg m^−2^, and decreased RS in the right lung. He had experienced coronary artery bypass grafting 12 years ago. Daily consumption of ASA 80 mg, warfarin 5 mg, atorvastatin 40 mg, and opium addiction were noted. Therefore, he underwent subcutaneous UFH 5000 IU TID in addition to continuation of his drugs. On the 4th day of admission, we were called for patient’s dyspepsia and lack of oral intake tolerance. Additionally, we found that he had nausea during last week, obstipation for last 2 days, and vomiting since a day before. Generalized abdominal distension, hypoactive bowel sounds, and a soft but tender abdomen in palpation were found. ECG showed postoperative changes and was suspicious for atrial fibrillation. The Hb was 9.2 g dL^−1^, INR > 4, PTT100, and Cr 2.4 mg dL^−1^. The abdominopelvic CT study with oral contrast revealed small bowel loop distension due to hyperaeration, long segment circumferential jejunal wall thickness secondary to edema, transitional zone in terminal ileum, and moderate free intraperitoneal fluid in line with high-grade bowel obstruction. Finally, according to patient’s condition he underwent therapeutic laparotomy with the clinical diagnosis of mesenteric ischemia on the same day. In the operating room we observed diffused mesenteric and wall hematoma of small bowel. Hence, we made no extra manipulation and patient was candidate for second look laparotomy. Despite receiving ICU care, he died the day after surgery. Autopsy study ruled out presence of DIC and/or SIC asa reason for expiration.

### Case 7

A 74-year-old SARS-CoV-2 confirmed woman with positive history of controlled hypertension and right-sided renal atrophy. She claimed of productive cough with fever and chill from 2 days ago. Clinical examination showed BP 100/70 mmHg, HR 88/min, RR 29/min, OT 41 °C, SO_2_ 85% in room air, BMI 30.1 kg m^−2^, and coarse crackle in the left basilar region. She suffered from the severe COVID-19 pneumonia and therefore admitted to ICU unit. Sixty milligram enoxaparin was prescribed two times a day in combination with daily ASA 80 mg. On the day 5th of admission, we were called to visit the patient due to abdominal distention and pain. Clinical findings manifested unstable hemodynamic values, decreased BS, generalized abdominal tenderness, and rebound tenderness in the left side compatible with acute peritoneal irritability. Bedside abdominal US study revealed a large heterogeneous hypoechoic 170 × 94 × 92 mm (about 730 cc) cystic hematoma in the left retroperitoneal flank region which shifted left kidney to the right. Hemoglobin level fell down from 13.1 to 6.3 g dL^−1^. Patient acutely underwent 2 units PBC and 4 units FFP perfusion and was then transferred to the operating room for exploratory laparotomy and hemostasis reestablishment. We drained near 1200 cc blood from the left retroperitoneal space intraoperatively. Hematoma was extended to mesenteric and bowel wall of splenic flexure, descending, and sigmoid colon. A closed suction drain was placed in left paracolic gutter and patient was referred back to ICU after the operation was finished. Next she totally received 6 units of the PBC and 10 units of the FFP and finally on postoperative date 4th her heart was arrested and cardiopulmonary resuscitation was unsuccessful. Autopsy study was otherwise normal except for presence of pulmonary involvement with COVID-19 and aforementioned bleeding.

### Case Summary

A brief report of aforementioned study subjects is presented in below Table 2.

**Table 2:** Study cases summary

Case	BMI (kg/m^2^)	Disease severity	Admitted to	Anticoagulant			Complication		Expiration date^+^
				Regiment	Total received days	Total received dosage	Type	Management	
1–26 years old female	24	Critical	ICU	Enoxaparin + ASA	5	400 mg + 400 mg	Right RSH^*^(70 cc)	Conservative	Expired in 10 days
2–70 years old female	32.3	Severe	ICU	UFH + ASA	6	9 × 10^4^ units + 480 mg	Right RSH (60 cc)	Conservative	Expired in 12 days
3–58 years old female	38.6	Moderate	Ward	UFH + ASA	2	3 × 10^4^ units + 160 mg	Right arm hematoma (40 cc)	Conservative	Expired in 7 days
4–51 years old female	34.7	Critical	ICU	Enoxaparin + ASA	7	840 mg + 560 mg	Left RSH extended to pelvic fossa (382 cc)	PBC & FFP infusion	Expired in 6 days
5–44 years old female	28.5	Severe	Ward	Enoxaparin + ASA	6	360 mg + 480 mg	Left RSH (64 cc) and preperitoneal hematoma (347 cc)	Conservative	Expired in 6 days
6–72 years old male	27.4	Moderate	Ward	UFH + ASA + Warfarin	4	6 × 10^4^ units + 320 mg + 20 mg	Small bowel wall and mesentery hematoma	Laparotomy	Expired in 1 day
7-74 y/o female	30.1	Severe	ICU	Enoxaparin + ASA	5	600 mg + 400 mg	Left retroperitoneal (730 cc), small bowel wall and mesentery hematoma	PBC & FFP infusion+ Laparotomy	Expired in 4 days

*Rectus sheath hematoma + following surgical consult

## Discussion

Since the knowledge of COVID-19 pathophysiology has confirmed microthrombosis events in end organs therefore, concurrent anticoagulation prescription is recommended for patients to prevent or treat thromboembolic events. VTE, DVT, PTE, ACS, and stroke are reported to have association with SARS-CoV-2 [1, 2]. Available scientific reports showed arteriovenous thromboemboli (6.6%), PTE (2.4–35%), isolated lower limbs DVT (0.9–54%), proximal DVT (0.5–25%), major bleeding (2.7%), and mortality (8.7–44%) in patients with SARS-CoV-2 who received anticoagulants [1]. Enoxaparin, UFH, fondaparinux, aspirin, and direct oral anticoagulants (DOAC) including rivoraxaban, apixaban, and dabigatran are prescribed in different guidelines [1–6]. However, there is no general consensus on any guideline for the therapy in moderate to severe disease [3]. For outpatient setting, if the risk of VTE is low, guidelines do not advise neither anticoagulant nor antiplatelet administration [3, 5]. Unfortunately, it is neither clear that how is the incidence of hemorrhagic events among COVID-19 patients after recovery or discharge from hospital nor there is reliable knowledge on how long anticoagulant should be continued. Namely, studies generally implied on that just critically ill patients with SARS-CoV-2 are at high risk for bleeding event according to the IMPROVE hemorrhagic risk score [1]. Additionally, it seems attributed side effects of anticoagulation protocols are still neglected in patients with COVID-19. Prior studies among non-COVID patients showed clearly that the risk of VTE after hospital discharge remains high but this phenomenon has not confirmed for COVID-19 patients since now [8–19]. Nonetheless, some authors recommend continuation of antithrombotics extensively after recovery from the disease; however, neither duration nor dosage is explained [1, 20]. Opponents point to that since association between COVID-19 and SIC and/or DIC is not exactly proven, any anticoagulation approach is uncertain [1].

Regarding previous medical data, UFH is preferred among SARS-CoV-2 positive with concurrent renal failure, instable hemodynamic status, and pleural effusion because physician is able to reverse the drug effect by antidote if needed; while among the others, the choice of anticoagulant is LMWH since it limits the staff exposure to patient for administration of the drug [1].

Analysis shows that total in-hospital mortality is similar between anticoagulant receivers and who do not receive but in intubated ICU admitted patients anticoagulants increases survival from 9 to 21 days when administered for at least 7 days [5, 21, 22]; although still 27% of ICU admitted COVID-19 patients manifest VTE while they receive anticoagulants [5]. Evidence noted that anticoagulation protocol occasionally also has no positive impact (6–16%) but makes additional complications [21].

In current case series neither the type of anticoagulant nor the administered dosage made safety window to prevent complication (bleeding and hematoma formation) in patients with COVID-19. The latter may be due to combination of anticoagulant and antiplatelet therapy; however, it seems the results are at least partially attributed to the nature of the disease because the guideline was safe previously in non-COVID patients that it was adopted from. Advocates of antiplatelet therapy implied on that it could decrease mortality while opponents considered antiplatelet therapy in critically or acutely ill hospitalized patients should be prevented [1, 6]. Regarding current study results, it seems prescription of any adjuvant anticoagulation protocol in patients with COVID-19 should be limited to selected patient especially in patients with severe disease. It is not yet clear how the SARS-CoV-2 primarily disturbs coagulation pathway and how it interacts when anticoagulants administered secondarily. Anyhow, according to available data severe COVID-19 disease is accompanied with coagulopathy disorders, and critically ill patients who are susceptible for thrombotic events including VTE and microembolization would benefit from thromboprophylaxis [1]. In addition, there is no agreement on anticoagulation guideline considering the dose or the duration of prescription [1–6]. In this study, all cases died due to bleeding while expiration was not expected with such volume of hemorrhage if the patient was a non-COVID. We found no definite evidence for DIC or SIC confirmation and patients except for hemorrhage and primary pulmonary involvement with SARS-CoV-2 were otherwise normal in autopsy studies. We hypothesized that presence of SARS-CoV-2 is associated with susceptibility to remarkable organ intolerance to circulation upset following even moderate bleeding events. Regarding aforementioned and our findings due to the nature of the COVID-19 syndrome vulnerable hemorrhagic outcome is probable despite that thromboembolic events are expected. Therefore, firstly therapeutic dose of anticoagulants with/without antiplatelet is not recommended in any patient with COVID-19 if no active thrombotic event is confirmed. Secondly, since there is no long-term prospective data available to clarify how hemorrhagic side effects would appear in patients with COVID-19 who receive prophylaxis regimen of anticoagulant it is advised to prescribe minimal sufficient dose of anticoagulant with no additional blood thinner agent.

## Conclusions

Since the VTE is pathophysiologically confirmed as a component in acute phase of COVID-19 syndrome particularly in critically ill patients, antithrombotic prescription is of advantage. However, the latter is generally accepted but there is no consensus on details. Because of unknown nature of SARS-CoV-2, possibility of life-threatening hemorrhagic phenomena independently from presence of DIC/SIC, and also uncertainty of outcome following anticoagulation administration in patients with COVID-19 it is highly recommended to use anticoagulants cautiously in selected subjects with confirmed thromboembolic event. Additionally utilization of anticoagulant should be restricted to minimal sufficient dose without any adjuvant blood thinner.

## Data Availability

The data used to support findings of this study is available in medical file archive unit of Beheshti Hospital, Kashan, Iran.
